# Two Novel Tyrosinase Inhibitory Sesquiterpenes Induced by CuCl_2_ from a Marine-Derived Fungus *Pestalotiopsis* sp. Z233

**DOI:** 10.3390/md11082713

**Published:** 2013-08-02

**Authors:** Bin Wu, Xiaodan Wu, Min Sun, Minhui Li

**Affiliations:** 1 Ocean College, Zhejiang University, Hangzhou 310058, China; 2 GEOMAR Helmholtz-Zentrum für Ozeanforschung Kiel, Kiel 24105, Germany; E-Mail: msun@geomar.de; 3 Center of Analysis and Measurement, Zhejiang University, Hangzhou 310058, China; E-Mail: wxd_zju@163.com; 4 Pharmacy Department, Baotou Medical College, Baotou 014060, China

**Keywords:** sesquiterpenes, *Pestalotiopsis* sp., abiotic stress, tyrosinase inhibitory activities

## Abstract

Two new sesquiterpenes, 1β,5α,6α,14-tetraacetoxy-9α-benzoyloxy-7β*H*-eudesman-2β,11-diol (**1**) and 4α,5α-diacetoxy-9α-benzoyloxy-7β*H*-eudesman-1β,2β,11,14-tetraol (**2**), were produced as stress metabolites in the cultured mycelia of *Pestalotiopsis* sp. Z233 isolated from the algae *Sargassum horneri* in response to abiotic stress elicitation by CuCl_2_. Their structures were established by spectroscopic means. New compounds **1** and **2** showed tyrosinase inhibitory activities with IC_50_ value of 14.8 µM and 22.3 µM.

## 1. Introduction

Marine-derived fungi, living in a stressful habitat, are of great interest as new promising sources of biologically active products. Since marine organisms live in a biologically competitive environment with unique conditions of pH, temperature, pressure, oxygen, light, nutrients and salinity, the chemical diversity of the secondary metabolites from marine fungi is considerably high [1,2,3,4,5,6]. New strategies of discovery of novel bioactive compounds including biotic [7,8,9] and abiotic [10] stress elicitations have been applied. Fungi of the genus *Pestalotiopsis* are characterized by their extensive distribution and wide genetic, biological and chemical diversity [11]. Natural products from the *Pestalotiopsis* species exhibit considerable chemical diversity and various bioactivities [11,12,13,14,15,16]. In this study, two novel sesquiterpenes were produced as stress metabolites in the cultured mycelia of *Pestalotiopsis* sp. Z233 isolated from algae *Sargassum horneri* in response to abiotic stress elicitation by CuCl_2_.

Tyrosinase is a multifunctional copper-containing enzyme, which catalyzes the hydroxylation of l-tyrosine to 3,4-dihydroxyphenylalanine (l-DOPA) and the subsequent oxidation of l-DOPA to dopaquinone, is widely distributed in microorganisms, animals and plants [17]. Tyrosinase inhibitors can be clinically useful for the treatment of some dermatological disorders associated with melanin hyperpigmentation [18]. Several tyrosinase inhibitors have been studied in our previous studies [19]. In continuation of our search for bioactive natural products that can be used for the treatment of dermatological disorders associated with melanin hyperpigmentation, stress metabolites in the cultured mycelia of *Pestalotiopsis* sp. Z233 were investigated.

## 2. Results and Discussion

The *Pestalotiopsis* sp. Z233, isolated from algae *Sargassum horneri*, was grown in the absence and, in parallel experiment, in the presence of the abiotic stress agent, CuCl_2_. Upon comparison of TLC plates of mycelial extracts from both conditions, two additional spots in the extract of CuCl_2_ treated culture were found. These two new compounds were presumably produced in response to abiotic stress. They were isolated by preparative TLC, and purified by Sephadex LH-20 column chromatography.

**Figure 1:**
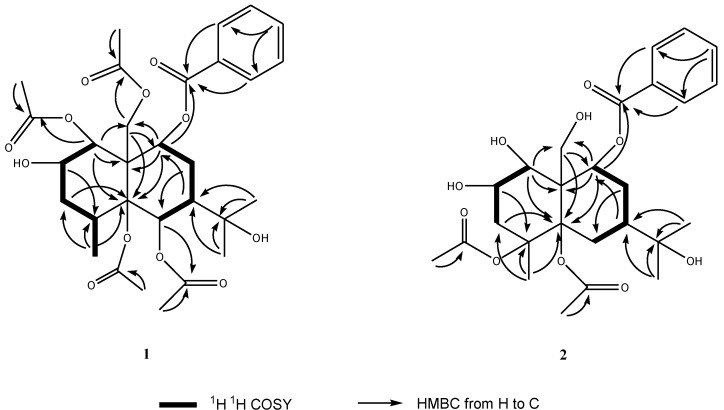
Key ^1^H ^1^H COSY and HMBC correlations of compounds **1** and **2**.

**Figure 2:**
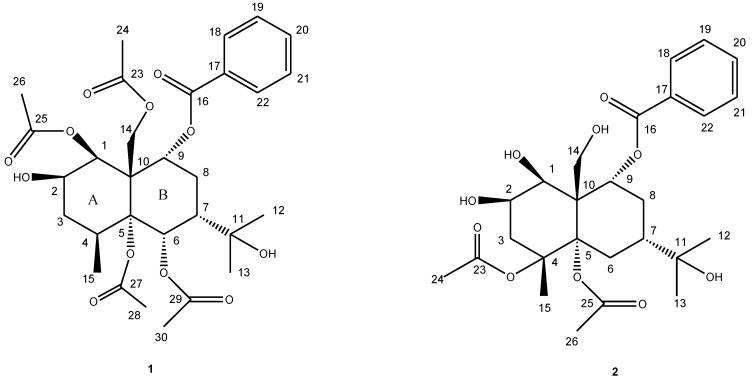
Structures of compounds **1** and **2**.

Compound **1** was obtained as a yellowish oil. The HR-TOF-MS exhibited an ion peak at *m/z* 593.2595 [M + H]^+^ (calcd. for C_30_H_41_O_12_, 593.2593), corresponding to the molecular formula, C_30_H_40_O_12_. The ^13^C NMR spectrum showed the presence of eight signals for four acetoxyl moieties, seven signals for a benzoyloxyl moiety, with the remaining 15 resonances corresponding to a sesquiterpene skeleton. The ^1^H and ^13^C NMR spectra indicated compound **1** to be a highly oxygenated eudesmane derivative (Table 1). The 15 signals for the eudesmane backbone comprised three methyls (δ_C_ 25.5, 30.1 and 17.3), one oxymethylene (δ_C_ 64.4), two methylenes (δ_C_ 30.3 and 34.2), two methines (δ_C_ 32.5 and 47.9), four oxymethines (δ_C_ 70.9, 68.5, 76.9 and 68.8), a quaternary carbon (δ_C_ 52.7), two oxygenated quaternary carbons (δ_C_ 88.8 and 82.4). Two of the three methyls (δ_C_ 25.5 and 30.1) were assigned to an oxygenated isopropyl group (carbinol signal at δ_C_ 82.4), with the third (δ_C_ 17.3) being Me-15. In the COSY spectrum of **1** (Figure 1), the oxymethine proton at δ_H_ 5.38 (m, H-2) was coupled with the oxymethine proton at δ_H_ 5.50 (d, *J* = 3.5 Hz, H-1) and methylene protons at δ_H_ 1.75 (dd, 14.5, 2.0 Hz, H-3a) and 2.13 (dd, 14.5, 3.2 Hz, H-3b). The methine proton at δ_H_ 2.22 (m, H-4) exhibited cross peaks with methylene protons of H_2_-3 and methyl protons at δ_H_ 1.12 (d, *J* = 7.5 Hz, Me-15) in the COSY spectrum of **1**. A sequence of H-1/H-2/H-3/H-4/Me-15 was deduced from above ^1^H ^1^H COSY analyses. Another sequence of H-6/H-7/H-8/H-9 was inferred from the observation of COSY cross peaks from the methine proton at δ_H_ 2.19 (m, H-7) to the oxymethine proton at δ_H_ 5.88 (d, *J* = 1.0 Hz, H-6) and methylene protons at δ_H_ 2.40 (m, H-8a) and 2.16 (m, H-8b), and cross peaks from H_2_-8 to the oxymethine proton at δ_H_ 5.34 (m, H-9). The benzoyloxyl moiety was assigned at C-9 from the observation of HMBC correlations from the oxymethine proton at δ_H_ 5.34 (m, H-9) and aromatic protons at δ_H_ 7.90 (dd, *J* = 8.0, 2.0 Hz) to the benzylic ester carbon resonance at δ_C_ 164.4 (s, C-16) and the oxygenated methylene carbon resonance at δ_C_ 64.4 (t, C-14). The HMBC peaks from two methyl groups at δ_H_ 1.41 (s, Me-12) and 1.42 (s, Me-13) to two oxygenated quaternary carbons at δ_C_ 88.8 (s, C-7) and 82.4 (s, C-11) positioned the oxygenated isopropyl group at C-7 of ring B. Three of four acetoxyls were assigned to C-1, C-6 and C-14 from analysis of the HMBC cross peaks of H-1/C-25, H-6/C-29 and H-14/C-23. The NOESY correlations from acetoxyl Me-28 to H-1 and H-2 at ring A positioned the remaining acetoxyl group at oxygenated C-5. The relative configuration of **1** was determined from the analyses of NOESY data. The oxygenated H-9 showed NOESY correlations with H_2_-14, indicating that benzoyloxyl moiety was α-oriented. The H-6 and H-7 protons at δ_H_ 5.88 (m) and 2.19 (m) showed NOESY correlations with the methylene protons at δ_H_ 5.00 and 4.25 (d, *J* = 12.7, H_2_-14), contributing an α-oriented acetoxyl unit and an α-oriented oxygenated isopropyl group in **1** as drawn. The NOESY cross peak of H_2_-14β/Me-15 implied a β-oriented CH_3_ at C-4. H-1 showed strong NOESY cross peak with aromatic H-18/22, revealing a β-oriented OH at C-1. The 3.5 Hz coupling constant between H-1 and H-2 indicated an equatorial H-2 proton, assigning the hydroxyl group at C-2 as a β configuration. This inference was also confirmed by the observation of the NOESY correlation from the axial proton of H-3α to H-2α. Therefore, the structure of this isolate was elucidated as 1β,5α,6α,14-tetraacetoxy-9α-benzoyloxy-7β*H*-eudesman-2β,11-diol (Figure 2). The assignment of NMR signals of **1** is listed in Table 1. 

**Table 1:** NMR Data (500 MHz) for Compound **1** and **2** in DMSO-*d*_6_.

Position	1		2	
	δ_C_ *^a^*^,*b*^, mult.	δ_H_ *^c^*, mult. (*J* in Hz)	δ_C_ *^a^*^,*b*^, mult.	δ_H_ *^c^*, mult. (*J* in Hz)
1	70.9, CH	5.50 d (3.5)	70.9, CH	5.50 d (3.5)
2	68.5, CH	5,38, m	68.4, CH	5.36, m
3a	30.3, CH_2_	1.75, dd (14.5, 2.0)	39.0, CH_2_	1.83 dd (14.0, 2.0)
3b		2.13, dd (14.5, 3.2)		2.09, dd (14.0, 3.0)
4	32.5, CH	2.22, m	68.8, C	
5	88.8, C		88.8, C	
6a	76.9, CH	5.88, d (1.0)	30.7, CH_2_	2.18, m
6b				1.80, m
7	47.9, CH	2.19, m	42.4, CH	2.06, m
8a	34.2, CH_2_	2.40, m	32.2, CH_2_	2.16, m
8b		2.16, m		2.02, m
9	68.8, CH	5.34, m	68.8, CH	5.69, m
10	52.7, C		50.9, CH	
11	82.4, C		83.1, C	
12	25.5, CH_3_	1.41, s	24.1, CH_3_	1.44, s
13	30.1, CH_3_	1.42, s	29.1, CH_3_	1.29, s
14a	64.4, CH_2_	5.00, d (12.7)	65.5, CH_2_	5.11, d (12.5)
14b		4.25, d (12.7)		4.79, d (12.5)
15	17.3, CH_3_	1.12, d (7.5)	26.0, CH_3_	1.37, s
16	164.4, C		164.6, C	
17	128.8, C		128.7, C	
18/22	129.5, CH	7.90, dd (8.0, 2.0)	129.5, CH	7.93, dd (8.0, 2.0)
19/21	128.5, CH	7.52, t (8.0)	128.5, CH	7.53, t (8.0)
20	133.6, CH	7.65, t (8.0)	133.5, CH	7.64, t (8.0)
23	169.9, C		168.3, C	
24	20.8, CH_3_	2.19, s	20.7, CH_3_	1.98, s
25	168.6, C		169.3, C	
26	20.0, CH_3_	1.46, s	20.0, CH_3_	1.38, s
27	169.4, C			
28	20.8 CH_3_	2.20, s		
29	169.6, C			
30	21.0, CH_3_	2.09, s		

*^a^* Recorded at 125 MHz; *^b^* Multiplicities inferred from DEPT and HMQC experiments; *^c^* Recorded at 500 MHz.

Compound **2** was obtained as a yellow gum. The HR-TOF-MS exhibited an ion peak at *m/z* 509.2385 [M + H]^+^ (calcd. for C_26_H_37_O_10_, 509.2381), indicating that the molecular formula was C_26_H_36_O_10_ with nine degrees of unsaturation. The NMR data (Table 1) in the downfield region of **2** were similar to those of the compound **1**, 1β,5α,6α,14-tetraacetoxy-9α-benzoyloxy-7β*H*-eudesman-2β,11-diol, which was isolated from the same fungus. However, the deshielded oxymethine proton of H-6 was absent in **2**. In the upfield region of the NMR spectra of **2**, two methylene protons at δ_H_ 2.18 and 1.80 (2 m, H_2_-6) were added, whereas two methyl were absent (Table 1), when compared with those of compound **1**. The doublet Me proton signal was downshifted to δ_H_ 1.37, and showed itself as a singlet in the upfield region of the NMR spectra of **2** as compared with that of compound **1**. These differences suggested that C-4 was substituted in **2**,and two acetoxyl groups were transformed to hydroxyl groups. No long range correlations were observed from oxymethine protons of H-1 and H-2, oxymethylene protons of H_2_-14 to any of the two acetoxyl ester carbonyl carbons, indicating that C-1, C-2 and C-14 were hydroxylated. The oxygenated quaternary carbon at δ_C_ 68.8 (s) was attributed to C-4 from the observation of long range correlations from the oxymethine proton signal at δ_H_ 5.36 (m, H-2) and the singlet Me proton signal at δ_H_ 1.37 (s, Me-15) to the carbon signal at δ_C_ 68.8 (s) (Figure 1). NOESY correlations from two acetoxyl Me proton signals to H-1 and H-2 at ring A positioned two acetoxyls at C-4 and C-5. The oxygenated isopropyl group was assigned at C-7 from the analyses of HMBC cross peaks of Me-12/C-7, M-13/C-7 and H-9/C-7. C-7 was proved to be linked to oxygenated quaternary carbon C-5 via a methylene bridge from analysis of the HMBC cross peaks from H_2_-6 and H-7 to C-5. The relative configuration of **2** was deduced to be the same as in **1**. The NOESY cross peak of Me-26/H-1α, Me-15β/H_2_-14β implied a *trans* eudesmane. The hydroxyl groups at C-1 and C-2 were β-oriented as shown by the observation of NOESY cross peaks of H-1α/H-3α and H-2α/H-3α in the NOESY experiment. H-7 and H-9 were proved to be β-oriented from the analyses of NOESY peaks of H_2_-14β/H-7β and H_2_-14β/H-9β. The new eudesmane derivative was elucidated as 4α,5α-diacetoxy-9α-benzoyloxy-7β*H*-eudesman-1β,2β,11,14-tetraol. The assignment of NMR signals of **2** is listed in Table 1. 

Two new compounds were tested for tyrosinase inhibitory activities [19]. 1β,5α,6α,14-tetraacetoxy-9α-benzoyloxy-7β*H*-eudesman-2β,11-diol and 4α,5α-diaacetoxy-9α-benzoyloxy-7β*H*-eudesman-1β,2β,11,14-tetraol showed IC_50_ values of 14.8 µM and 22.3 µM, when the active compounds were compared to the standard tyrosinase inhibitor kojic acid (IC_50_ = 21.2 µM). 

## 3. Experimental Section

### 3.1. General Experimental Procedures

Optical rotations were recorded on a Perkin-Elmer-341 polarimeter. The IR spectra (CHCl_3_) were run on a NicoletAvatar-360FT-IR spectrometer. ^1^H NMR (500 MHz) and ^13^C NMR (125 MHz) spectra were measured at 25 °C on a Bruker AVANCE DMX 500 NMR spectrometer with TMS as internal standard. TOF-MS were recorded on a GCT-Premier GC-TOF-MS spectrometer. ESIMS were recorded on an Agilent 6460 Triple Quad LC/MS. TLC was performed using Merck precoated plates (Silica gel 60 F254) of 0.25 mm thickness. Sephadex LH-20 (Amersham) was used for column chromatography.

### 3.2. Fungal Cultivation and Stress Applications

The fungus *Pestalotiopsis* sp. Z233 was separated from the marine algae *Sargassum horneri*, which was collected from seashore in Wenzhou, China, and identified by its ITS-5.8s rDNA sequences. Subcultures of the organism are deposited at the Department of Ocean Science and Engineering, Zhejiang University. Cultures were separated into control (5 L) and stressed groups (5 L). The control fungus was grown in the culture medium consisting of (g·L^−1^) sucrose (66.0 g), yeast extract (10.0 g), silkworm chrysalis (30.0 g), MgSO_4_·7H_2_O (0.4 g), and KH_2_PO_4_ (0.4 g) in sterilized and filtrated natural seawater collected from Wenzhou, China. The stressed culture medium consisted of additional 50 µmol/L CuCl_2_. The fermentations were carried out at 24 °C for 10 days. 

### 3.3. Extraction and Isolation

The whole culture of the control and stressed broth of *Pestalotiopsis* sp. Z233 (5 L) were filtered. The air-dried mycelia of control (48.4 g) and the stressed group (45.4 g) were extracted at room temperature with MeOH (3 × 1 L), respectively. The extracts were evaporated in *vacuo* to afford a gummy residue for both Cu treated (2.2 g) and corresponding control (2.7 g). The residues were partitioned in H_2_O (500 mL) and extracted successively with EtOAc (3 × 500 mL) and *n*-butanol (3 × 500 mL). The EtOAc and *n*-butanol extracts of the treated and control cultures were subjected to TLC examination on aluminium sheets pre-coated with Silica gel 60 F 254 (Merck). The spots were applied in as equal amounts as possible. The plates were developed in the following developing solvent systems: benzene–acetone (6:1), benzene–EtOAc (5:1) petroleum ether–EtOAc (5:1) for the EtOAc extract; CHCl_3_–MeOH (3:1), CH_2_Cl_2_–MeOH (4:1) and benzene–CHCl_3_–MeOH (1:3:1) for the *n*-butanol extract. After development, the plates were examined under UV light (250 nm) to locate any additional spots in the different extracts of the treatments in comparison with those of the corresponding control extracts. The spots on the plates were also visualized by spraying with an EtOH–H_2_SO_4_ solution. Two additional compounds were detected on the plates developed in benzene–acetone (6:1) solvent system in the EtOAc extract of stress elicited mycelium. Several prep-TLC plates were prepared and the target compounds were isolated by preparative TLC in the developing solvent systems of benzene–acetone (6:1). The crude compounds were applied to a Sephadex LH-20 column (1 × 80 cm, 38 g, Amersham), and eluted with acetone to yield pure compounds **1** (8.4 mg) and **2** (7.0 mg).

1β,5α,6α,14-Tetraacetoxy-9α-benzoyloxy-7β*H*-eudesman-2β,11-diol (**1**): yellowish oil; [α]^24^_D_ −22 (*c* 0.001, CHCl_3_); UV (MeOH) λ_max_ (log є) 230 (4.11), 254 (4.23), 280 (4.56) nm; IR ν_max_ 3422, 1766, 1614, 1515, 1333, 1111, 721 cm^−1^; ^1^H NMR and ^13^C NMR, see Table 1; ESIMS *m/z* 593 [M + H]^+^; HR-TOF-MS *m/*z 593.2595 [M + H]^+^ (calcd. for C_30_H_41_O_12_, 593.2593). 

4α,5α-Diacetoxy-9α-benzoyloxy-7β*H*-eudesman-1β,2β,11,14-tetraol (**3**): yellow gum; [α]^24^_D_ −26 (*c* 0.001, CHCl_3_); UV (MeOH) λ_max_ (log є) 230 (4.30), 254 (4.17), 280 (4.43) nm; IR ν_max_ 3416, 1766, 1615, 1513, 1324, 1113, 824 cm^−1^; ^1^H NMR and ^13^C NMR, see Table 1; ESIMS *m/z* 509 [M + H]^+^; HR-TOF-MS *m/*z 509.2385 [M + H]^+^ (calcd. for C_26_H_37_O_10_, 509.2381). 

### 3.4. Tyrosinase Inhibition Assay

Tyrosinase inhibition assay were performed in 96-well microplate format using SpectraMax 340 microplate reader according to the previously developed method [19]. The compounds were initially screened for the *O*-diphenolase inhibitory activity of tyrosinase using l-DOPA as substrate. All active inhibitors from the preliminary screening were subjected to IC_50_ studies. Compounds were dissolved in methanol to a concentration of 2.5%. Mushroom tyrosinase (28 nM) were preincubated with the compounds in 50 nM Na-phosphate buffer (pH 6.8) for 10 min at 25 °C. Then the l-DOPA (0.5 mM) was added to the reaction mixture and the enzyme reaction was monitored by measuring the change in absorbance at 475 nm (at 37 °C) due to the formation of the DOPAchrome for 10 min. The percent inhibition of the enzyme was calculated as follows:

Percent inhibition (%) = [(*B* − *S*)/*B*] × 100

The *B* and *S* are the absorbance for the blank and samples, respectively. After screening of the compounds, median inhibitory concentration (IC_50_) was calculated. Kojic acid was used as standard inhibitors for the tyrosinase. 

## 4. Conclusions

In this study, two novel sesquiterpenes were produced as stress metabolites by the mycelia of cultured *Pestalotiopsis* sp. Z233 in response to abiotic stress elicitation by CuCl_2_. These new metabolites possess interesting eudesmane skeleton, and exhibited potent tyrosinase inhibitory activities, indicating that metal stress elicitation on marine fungi was a promising strategy to discover new bioactive natural compounds.

## References

[1] Debbab A., Aly A.H., Lin W.H., Proksch P. (2010). Bioactive compounds from marine bacteria and fungi. Microb. Biotechnol..

[2] Saleema M., Ali M.S., Hussain S., Jabbar A., Ashraf M., Lee Y.S. (2007). Marine natural products of fungal origin. Nat. Prod. Rep..

[3] Yamazaki H., Rotinsulu H., Kaneko T., Murakami K., Fujiwara H., Ukai K., Namikoshi M. (2012). A new dibenz[*b*,*e*]oxepine derivative, 1-hydroxy-10-methoxy-dibenz[*b*,*e*]oxepin-6,11-dione, from a marine-derived fungus, *Beauveria bassiana* TPU942. Mar. Drugs.

[4] Sun L., Li D., Tao M., Chen Y., Dan F., Zhang W. (2012). Scopararanes C–G: New oxygenated pimarane diterpenes from the marine sediment-derived fungus *Eutypella scoparia* FS26. Mar. Drugs.

[5] Bhadury P., Mohammad B.T., Wright P.C. (2006). The current status of natural products from marine fungi and their potential as anti-infective agents. J. Ind. Microbiol. Biotechnol..

[6] Zhang L., An R., Wang J., Sun N., Zhang S., Hu J., Kuai J. (2005). Exploring novel bioactive compounds from marine microbes. Curr. Opin. Microbiol..

[7] Jensen P.R., Fenical W. (1994). Strategies for the discovery of secondary metabolites from marine bacteria: Ecological perspectives. Annu. Rev. Microbiol..

[8] Cueto M., Jensen P.R., Kauffman C., Fenical W., Lobkovsky E., Clardy K. (2001). Pestalone, a new antibiotic produced by a marine fungus in response to bacterial challenge. J. Nat. Prod..

[9] Park H.B., Kwon H.C., Lee C.-H., Yang H.O. (2009). Glionitrin A, an antibiotic−antitumor metabolite derived from competitive interaction between abandoned mine microbes. J. Nat. Prod..

[10] Wu F., Jiang W., Wu B. (2013). Methodological aspects about determination of plant defensive phenolics in response to stress. Curr. Anal. Chem..

[11] Xu J., Ebada S.F., Proksch P. (2010). *Pestalotiopsis*, a highly creative genus: Chemistry and bioactivity of secondary metabolites. Fung. Divers..

[12] Wei M.-Y., Li D., Shao C.L., Deng D.-S., Wang C.Y. (2013). (±)-Pestalachloride D, an antibacterial racemate of chlorinated benzophenone derivative from a soft coral-derived fungus *Pestalotiopsis* sp. Mar. Drugs.

[13] Strobel G., Yang X., Sears J., Kramer R., Sidhu R.S., Hess W.M. (1996). Taxol from *Pestalotiopsis microspora*, an endophytic fungus of *Taxus wallachiana*. Microbiology.

[14] Liu L., Liu S., Chen X., Guo L., Che Y. (2009). Pestalofones A–E, bioactive cyclohexanone derivatives from the plant endophytic fungus *Pestalotiopsis fici*. Bioorg. Med. Chem..

[15] Liu L., Liu S., Jiang L., Chen X., Guo L., Che Y. (2008). Chloropupukeananin, the first chlorinated pupukeanane derivative, and its precursors from *Pestalotiopsis fici*. Org. Lett..

[16] Liu L., Bruhn T., Guo L., Götz D.C.G., Brun R., Stich A., Che Y., Bringmann G. (2011). Chloropupukeanolides C–E: Cytotoxic pupukeanane chlorides with a spiroketal skeleton from *Pestalotiopsis fici*. Chem. Eur. J..

[17] Vamos-Vigyazo L. (1981). Polyphenol oxidase and peroxidase in fruits and vegetables. Crit. Rev. Food Sci. Nutr..

[18] Kim Y.-J., Uyama H. (2005). Tyrosinase inhibitors from natural and synthetic sources: Structure, inhibition mechanism and perspective for the future. Cell. Mol. Life Sci..

[19] Wu B., Zhang X., Wu X. (2012). New lignan glucosides with tyrosinase inhibitory activity from exocarp of *Castanea henryi*. Carbohydr. Res..

